# Environmental Factors Impacting Bone-Relevant Chemokines

**DOI:** 10.3389/fendo.2017.00022

**Published:** 2017-02-14

**Authors:** Justin T. Smith, Andrew D. Schneider, Karina M. Katchko, Chawon Yun, Erin L. Hsu

**Affiliations:** ^1^Department of Orthopaedic Surgery, Northwestern University Feinberg School of Medicine, Chicago, IL, USA; ^2^Simpson Querrey Institute for BioNanotechnology, Northwestern University, Chicago, IL, USA

**Keywords:** chemokines, bone healing, environmental toxins, dioxin, metal, endocrine disruptors, volatile organic compound

## Abstract

Chemokines play an important role in normal bone physiology and the pathophysiology of many bone diseases. The recent increased focus on the individual roles of this class of proteins in the context of bone has shown that members of the two major chemokine subfamilies—CC and CXC—support or promote the formation of new bone and the remodeling of existing bone in response to a myriad of stimuli. These chemotactic molecules are crucial in orchestrating appropriate cellular homing, osteoblastogenesis, and osteoclastogenesis during normal bone repair. Bone healing is a complex cascade of carefully regulated processes, including inflammation, progenitor cell recruitment, differentiation, and remodeling. The extensive role of chemokines in these processes and the known links between environmental contaminants and chemokine expression/activity leaves ample opportunity for disruption of bone healing by environmental factors. However, despite increased clinical awareness, the potential impact of many of these environmental factors on bone-related chemokines is still ill defined. A great deal of focus has been placed on environmental exposure to various endocrine disruptors (bisphenol A, phthalate esters, etc.), volatile organic compounds, dioxins, and heavy metals, though mainly in other tissues. Awareness of the impact of other less well-studied bone toxicants, such as fluoride, mold and fungal toxins, asbestos, and chlorine, is also reviewed. In many cases, the literature on these toxins in osteogenic models is lacking. However, research focused on their effects in other tissues and cell lines provides clues for where future resources could be best utilized. This review aims to serve as a current and exhaustive resource detailing the known links between several classes of high-interest environmental pollutants and their interaction with the chemokines relevant to bone healing.

## Introduction

Chemokines play an important role in normal bone physiology and the pathophysiology of many bone diseases. The recent increased focus on the individual roles of this class of proteins in the context of bone has shown that members of the two major chemokine subfamilies—CC and CXC—promote the formation of new bone and the remodeling of existing bone in response to a myriad of stimuli. These chemotactic molecules are crucial in orchestrating appropriate cellular homing, osteoblastogenesis, and osteoclastogenesis during normal bone repair. A recent review by Gilchrist and Stern provided a comprehensive assessment of the role key chemokines and receptors play in the regulation of bone healing (1). Several of these chemokines have received growing attention in recent literature and their mechanisms of action have been further defined.

Organizations and initiatives such as *Physicians for Social Responsibility* and the *Collaborative on Health and the Environment* recognize that environmental contaminants such as endocrine-disrupting chemicals (EDCs) are of serious concern with regard to bone health^1^ (2). A great deal of focus has been placed on environmental exposure to various endocrine disruptors (bisphenol A, phthalate esters, etc.), volatile organic compounds (VOCs), dioxins, and heavy metals. Awareness of the impact of other less well-studied bone toxicants, such as fluoride, mold and fungal toxins, asbestos, and chlorine, is also growing.

Bone healing is a complex cascade of carefully regulated processes, including inflammation, progenitor cell recruitment, differentiation, and remodeling. The extensive role of chemokines in these processes and the known links between environmental contaminants and chemokine expression and activity leaves ample opportunity for disruption of bone healing by environmental factors. However, despite increased clinical awareness, the potential impact of many of these environmental factors on bone-related chemokines is still ill defined. This review aims to serve as a current and exhaustive resource detailing the known links between several classes of high-interest environmental pollutants and their interaction with the chemokines relevant to bone healing. Areas where the literature is lacking and further research is prudent are also highlighted.

## Endocrine Disruptors

Endocrine-disrupting chemicals are of specific concern due to their exogenous influence over the endocrine system. These compounds exert their effects independent of biofeedback loops, leading to potentially harmful consequences. EDCs can be either natural or synthetic in origin. Natural EDCs consisting of organically produced compounds are out of the scope of this review and will not be discussed. Synthetic EDCs are commonly designed with another purpose in mind (e.g., pesticides or plasticizers), only to have their endocrine effects subsequently discovered. These effects have been observed as developmental anomalies in both invertebrate and aquatic species (3, 4). A summary of endocrine disruptors and their effects on bone-related chemokines is shown in Table 1.

**Table 1:** **Endocrine disruptors: chemokine changes**.

Substance	Chemokine(s) involved	Effect(s)	Cell/tissue type	Reference
BPA	CXCL2 CXCL4 CXCL12 CXCL14 CCL20	↓	Mouse mammary gland	Fischer et al. (5)

	CXCL12	↑	Human BG-1	Hall and Korach (6)

			Human MCF-7 and T47D	Habauzit et al. (7)

			Human ECC-1 and T47D	Gertz et al. (8)

BPAF	CXCL12	↑	Human T47D	Li et al. (9)

DEHP	*CXCL1* *CXCL2* *CXCL3* *CXCL6* *CCL3*	↑	Human THP-1	Nishioka et al. (10)

	CCL2	↓	Mouse hypothalamus tissue	Win-Shwe et al. (11)

DINP	CCL2	↓	Mouse hypothalamus tissue	Win-Shwe et al. (11)

PFAS	CCL2	No changes observed	Human serum	Stein et al. (12)
	CCL3			

*Known effects of endocrine disruptors on chemokines (BPA, bisphenol A; BPAF, bisphenol AF; DEHP, di-(2-ethylhexyl)-phthalate; DINP, di-isononyl-phthalate; PFAS, perfluoroalkyl substance)*.

### Bisphenol A

Bisphenol A (BPA) is a chemical compound that has been used in commercial plastics and epoxy resins since the 1950s. The widespread use of BPA in manufacturing has led to its ubiquitous presence in industrialized environments (13). The primary method of exposure in humans is through the diet, mainly through drinking water in industrialized regions, as well as from food and drink storage or use containers (14). Specifically, BPA can leach into foods from the plastics or resins used during the manufacturing of such containers (e.g., water bottles, food cans, etc.). Up to 90% of the US population has detectable blood levels of BPA, which has been a growing public health concern (15). Thus far, chronic exposure has been linked to cancer, metabolic disorders, and a range of reproductive and cardiovascular diseases (16, 17).

Bisphenol A functions as an endocrine disruptor through binding to ERα and ERβ with roughly 1/1,000th the affinity of estradiol (18, 19). Because estradiol can attenuate osteoclastogenesis and induce osteoblast differentiation (20, 21), Hwang et al. recently examined the effects of BPA on osteoclast formation and osteoblast differentiation *in vitro* (22). The authors found that BPA directly inhibited both osteoclastogenesis and osteoblastogenesis, and increased apoptosis in both types of progenitors. BPA suppressed RANK expression in differentiating osteoclasts and RunX2 and Osterix expression in preosteoblasts. Wnt/β-catenin signaling and bone nodule formation was also reduced. These authors demonstrate that BPA promotes apoptosis of osteoclasts and osteoblasts through MAPK cascades and death receptor pathways in a dose-dependent manner (22).

Despite the effects of BPA on osteoblasts and osteoclasts, our search criteria yielded no studies examining BPA-induced chemokine expression changes in osteogenic models. However, given its endocrine-disrupting status, several studies have examined its impact on chemokines in reproductive tissues such as mouse mammary, breast cancer, and ovarian cancer cells (5–8). Significant decreases in CXCL2, CXCL4, CXCL14, and CCL20 have been observed in these various tissues with results that suggest BPA may act through pathways independent of estrogen. Additionally, the related compound, Bisphenol AF (BPAF), has demonstrated upregulation of CXCL12 in T47D ERα-positive cancer cell lines (23). Considering the critical role of the CXCL12/CXCR4 axis on bone regeneration, the known effects of BPA and BPAF on tissues other than bone make clear the importance of evaluating their effects in the context of bone healing.

### Phthalate Esters

Phthalate esters comprise another group of EDCs that are used to increase the flexibility of polymers, such as polyvinyl chloride (PVC)-based products (e.g., toys, food wraps, medical devices, and flooring). Similar to BPA, humans are exposed to phthalates through ingestion, inhalation, and physical contact on a daily basis (24, 25). Two particular phthalates, di-(2-ethylhexyl) phthalate (DEHP) and di-isononyl phthalate (DINP), have been examined in the context of the chemokine expression and bone formation (10, 11).

DEHP is cytotoxic to neonatal rat calvarial osteoblasts at high doses (1,000 µM), while inducing proliferation at low doses (10 µM) (26). In that system, osteogenic differentiation was inhibited at high doses of DEHP, with reduced *RunX2* and ALP expression and decreased collagen and mineralization staining (26).

Inflammation is an important early step in the bone healing cascade. Chemokines play a critical role in recruitment of macrophages and progenitor cells to the site of injury, where they remove necrotic tissue and initiate the regenerative process. However, this process is tightly regulated, and chronic inflammatory diseases are associated with systemic bone loss (27). Even subclinical inflammation has been shown to increase fracture risk and alter bone remodeling (28). Nishioka et al. examined the effects of DEHP on the production of inflammatory cytokines in activated macrophage-like THP-1 cells. A 3-h exposure to 200 µM DEHP significantly induced *CXCL1, CXCL2, CXCL3, CXCL6*, and *CCL3* transcripts (10). PTH induces CXCL1 expression in osteoblasts, which then attracts osteoclasts *via* CXCR2 (29). Interestingly, CXCL1 acts as a chemoattractant for osteoclast precursors without promoting osteoclastic differentiation, acting as a “primer” for additional factors (29). That said, it is reasonable to speculate that increased CXCL1 expression by macrophages recruited during bone repair could also prove chemotactic for osteoclasts. Further research should examine whether DEHP influences CXCL1 secretion in osteoblasts in a similar manner.

Human osteoblasts express CCL2, which is an important chemoattractant for monocytes and macrophages (30). Additionally, CCL2 is critical for osteoclastogenesis, and its absence results in inhibition of osteoclast formation (31). In studies examining the hypothalami of DEHP- and DINP-treated male mice, Win-Shwe et al. observed decreases in *CCL2* and *TNF-*α expression in the DINP-exposed groups (11). Considering the important role that CCL2 plays on osteoclastogenesis, further investigation to the effects of DINP in bone models should be explored.

### Perfluoroalkyl Substances (PFASs)

Perfluoroalkyl substances have been widely used as protective coatings in water- and stain-resistant clothing, furnishings, and non-stick home goods for over 60 years (32). These chemicals have been classified as EDCs based on their hormonal and metabolic actions (33, 34). PFASs are ubiquitous in the environment, and detectable amounts are found in humans worldwide (35). Examination of the U.S. National Health and Nutritional Examination Survey (NHANES) from 1999 to 2008 showed that four PFASs were found in 95% of the U.S. population: perfluorooctanoic acid (PFOA), perfluorooctane sulfonic acid (PFOS), perfluorohexane sulfonic acid (PFHxS), and perfluorononanoic acid (PFNA) (36, 37). No research has been conducted to examine the effects of PFASs on chemokine production. However, there is evidence justifying further investigation into the mechanisms of PFAS action in osteogenic models. In a representative sample of the U.S. population collected from the NHANES 2009–2010, serum PFAS levels were inversely correlated with bone mineral density (BMD) of the femur and lumbar spine (32). Most of the associations were limited to women, despite men having higher serum PFAS levels. In general, postmenopausal women had stronger associations with lower BMD than their premenopausal counterparts. Osteoporosis was associated with exposure to PFOA, PFNA, and PFHxS in women (32).

### Organotins

Organotins are environmental contaminants considered to be obesogens because they activate peroxisome proliferator-activated receptor γ (PPARγ), the primary regulator of adipogenesis (38, 39). These compounds are broadly used as pesticides, catalytic agents, plastic stabilizers, and antifouling agents (40). The actions of organotins on skeletal development have been examined in animal models (41, 42). Tsukamoto et al. demonstrated that mouse fetuses exposed to tributyltin chloride (TBT) showed reduced calcification of the supraoccipital bone (41). *In vitro*, TBT dose-dependently inhibited differentiation of primary rat calvarial osteoblasts, with reduced ALP activity, mineral deposition rate, and osteocalcin expression levels.

Despite these data, few studies have been published examining the effects of organotins on chemokine expression. Schutte et al. examined chemokine expression after implanting an organotin-stabilized PVC cage implant in Sprague-Dawley rats (43). They demonstrated a long-term (56 days postimplantation) 10-fold increase of CCL3, a pro-inflammatory chemokine responsible for increasing osteoclast motility (44). CCL2 demonstrated a significant increase on postoperative day 1, but then returned to normal levels. Given the endocrine activity associated with these compounds and their known effects on skeletal development, the mechanisms by which this class of compounds affects osteogenesis should be clarified.

## Volatile Organic Compounds

In much of the industrialized world, individuals spend most of their time indoors. Indoor organic contaminants are classified by their volatility, dubbing them VOCs. These compounds are released from paints, adhesives, construction materials, cleaners, tobacco smoke, and carpets (45, 46). Because of the numerous sources found indoors, many of these compounds are present at concentrations significantly higher than outside (47). Elevated concentrations of VOCs are associated with asthma and respiratory diseases in children and adults (48–52). Table 2 summarizes the effects of the VOCs described below on chemokines involved in bone repair.

**Table 2:** **Volatile organic compounds (VOCs): chemokine changes**.

Substance	Chemokine(s) involved	Effect(s)	Cell/tissue type	Reference
Benzene	CXCL12	↑ 1 h after exposure to 1,4-benzoquinone	Human mesenchymal stem cell	Zolghadr et al. (53)
		↓ 24 h after exposure to hydroquinone		

	CXCL8 CCL3 CCL5 CCL11 CCL2	↑	Human PBMC and plasma	Gillis et al. (54)

Chlorobenzene	CCL2	↑ Indoor-relevant concentrations	Human PBMC and A549	Lehmann et al. (55)

			Human A549	Fischader et al. (56)

		↓ High concentrations	Human A549	Fischader et al. (56)

m-Xylene and styrene	CCL2	↑ Indoor-relevant concentrations	Human A549	Fischader et al. (56)
		↓ High concentrations		

Other aliphatic compounds	CXCL8 CCL2	No change	Human A549	Fischader et al. (56)

*Known effects of VOCs on chemokines. Other aliphatic compounds refer to: n-non-ane, n-decane, n-undecan, n-dodecane, n-tridecane, and methylcyclopentane*.

### Benzene

Benzene is a colorless, flammable organic liquid that can volatize to vapors at room temperatures. Used as an industrial chemical in the manufacturing of other compounds, it is also a component of crude oil, gasoline, and cigarette smoke (57, 58). Benzene was among the first compounds identified as a Group I known human carcinogen by the International Agency for Research on Cancer (59). The role of benzene in the development of acute myeloid leukemia and other myelodysplastic syndromes is well established (60–63). Human exposure to benzene occurs through inhalation and dermal absorption as well as ingestion of contaminated food and water (64). It is widely agreed that the toxicity of benzene results from its metabolism to reactive intermediates. In the liver, benzene is metabolized by CYP2E1 to phenol, which undergoes hydroxylation to hydroquinone, catechol, and 1,2,4-benzenetriol (65). Catechol and hydroquinone persist in bone marrow, where they are oxidized to 1,2-benzoquinone and 1,4-benzoquinone by myeloperoxidase (54, 66). The mechanism(s) by which these metabolites influence carcinogenesis have not been fully clarified (67).

While the effects of benzene and its metabolites on the immune and hematopoietic systems are well established, the influence of these compounds on bone development and skeletal remodeling is less fully understood. Zolghadr et al. examined the effects of benzene, hydroquinone, and 1,4-benzoquinone on human-derived mesenchymal stem cells (MSCs) (53). They examined cell viability, apoptosis, and expression of genes relevant to hematopoiesis and skeletal remodeling. At the lowest concentrations tested, all three compounds increased cell division rate after only 24 h, and the effects were attributed to cell cycle control defects (68). Interestingly, *RunX2* expression was significantly *up*regulated (up to eightfold) by all three chemicals, as was Wnt5a, which is a non-canonical Wnt ligand. Simultaneously, *Dkk1* expression was induced, which inhibits canonical Wnt signaling by interacting with the Wnt co-receptors, LRP-5/6. The authors hypothesized that the increase in *Dkk* expression was a response to induction of *RunX2* and canonical Wnt signaling.

CXCL12, along with its receptor, CXCR4, plays a critical role in mesenchymal and hematopoietic stem cell recruitment, as well as BMP-mediated osteoblastic differentiation (69, 70). A major regulator of BMSC growth, CXCL12 expression is thought to decrease acutely after osteoblastic lineage commitment (71, 72), suggesting that its role is critical in the early stages of osteogenesis (69). In human MSC, CXCL12 expression was upregulated after short-term (1 h) exposure to 1,4-benzoquinone, but was downregulated after 24 h exposure to hydroquinone. The dysregulation of the Wnt and CXCL12 signaling pathways could have pronounced implications on bone regeneration. Further research needs to be conducted to define the upstream mechanisms regulating the effects of benzene metabolites on the CXCL12/CXCR4 axis.

### Chlorobenzene

Chlorobenzene is one of the most widely used chlorinated benzenes (55). It functions as a solvent for many substances, such as paints, adhesives, waxes, and polishes, and it is also commonly used in the dry-cleaning industry (73). Chlorobenzene inhalation can lead to irritation of the eyes and respiratory tract, drowsiness, loss of coordination, CNS depression, and loss of consciousness (73, 74). Additionally, epidemiological studies suggest that exposure to chlorobenzene is associated with allergic sensitizations and Th2-primed T cell immunity (75).

Chlorobenzene has been shown to induce CCL2, a chemokine critical for osteoclast formation, production at indoor-relevant concentrations in TNF-α-primed alveolar A549 cells (55, 56). Additional research has shown that the mechanism through which chlorobenzene induces CCL2 is through the NF-kB and MAPK pathways (76). Despite the CCL2 connection, however, no studies have attempted to correlate exposure with impaired osteoclastic differentiation, osteogenesis, or bone healing.

### Xylene, Styrene, and Aliphatic VOCs

Similar to chlorobenzene, m-Xylene and styrene have been shown to induce CCL2 production, albeit in A549 cells. Still, dose-dependent alterations in osteoclast precursor recruitment and differentiation are worth investigating, which could lead to impaired bone remodeling and healing.

Fischader et al. additionally examined the effects of several aliphatic compounds (n-non-ane, n-decane, n-undecan, n-dodecane, n-tridecane, and methylcyclopentane) on A549 cells (56). Neither the single aliphatic compounds nor a mixture of the group demonstrated any effect on cytokine/chemokine release.

## Dioxins and Dioxin-Like Compounds

Dioxins and dioxin-like compounds (Benzo[a]pyrene, polychlorinated dibenzofurans, non-ortho-substituted, and mono-ortho-substituted pentachlorobiphenyls) compose a group of highly toxic environmental pollutants. Dioxins are formed as unintentional by-products of industrial manufacturing, when chlorine-based compounds are burned in the presence of hydrocarbons. Other important sources are waste-burning incinerators and backyard burning. 2,3,7,8-Tetrachlorodibenzo-*p*-dioxin (TCDD) was the major toxic contaminant in Agent Orange, the defoliant used extensively during the Vietnam War. Since dioxins and dioxin-like compounds are lipophilic, they bioaccumulate through the food chain. As such, the major source of dioxin exposure is the diet, the bulk coming from meat and dairy products, as well as fish. The half-life of TCDD is estimated as 7–10 years in humans.

TCDD causes a multitude of adverse health effects, including immune suppression, cancer (e.g., non-Hodgkin’s lymphoma, chronic lymphocytic leukemia, and multiple myeloma), reproductive and developmental toxicity, cognitive function, and skin disorders (77–79). It has been shown to inhibit cell migration and osteogenic differentiation *in vitro* (80), alter normal bone phenotype (80–87), and inhibit bone healing *in vivo* (85, 88, 89). TCDD primarily exerts its deleterious effects through the aryl hydrocarbon receptor (AhR) pathway (83). Upon activation, this receptor acts as a transcription factor to modulate the expression of a large battery of genes, such as the Cytochrome P450 1 (CYP1) family, members of which are responsible for the Phase I biotransformation of hydrophobic xenobiotics. The Ahr can work through genomic (*via* binding to dioxin response elements in promoter/enhancer regions of dioxin-responsive genes) or non-genomic means. A great deal of evidence suggests Ahr cross-talk with NFKB signaling (90, 91), estrogen receptor signaling, and hypoxia-inducible factors (92–95).

With regard to immunomodulation, emerging data link AhR expression/activation with chronic diseases such as degenerative arthritis. Ahr activation promotes secretion of inflammatory cytokines, leading to local bone loss, inhibition of osteoblast proliferation and differentiation, and causes osteoporosis in mice (81, 88). Studies also suggest that the AhR acts as a negative regulator of stem cell proliferation (96), which may play a role on monocyte recruitment during bone remodeling. AhR has been shown to induce Th17 cell differentiation through a miRNA-mediated process (97–100). Th17 cells play a major role in the pathology of rheumatoid arthritis (RA), and the Ahr has been implicated in RA development through this mechanism. The downstream effects of Ahr-induced immunomodulation are excessive osteoclastic differentiation and inflammation (93, 101–103). AhR activation has also been tied to a diminished capacity for bone regeneration and healing (89, 104). The notion of excessive osteoclast proliferation by AhR ligands is supported by the impaired osteoclastogenesis and increased bone mass that is seen in AhR knockout mice (83).

### TCDD (2,3,7,8-tetrachlorodibenzo-*p*-dioxin)

The most toxic and well-studied dioxin and AhR ligand is 2,3,7,8-tetrachlorodibenzo-*p*-dioxin (TCDD). TCDD was first shown to downregulate CXCL12 and CXCR4 expression in breast and ovarian cancer cells (23). A study conducted by Casado et al. demonstrated diminished CXCL12 migration of LSK cells exposed to TCDD with *increased* cell surface expression of CXCR4 (80). However, levels of *CXCR4* mRNA were not induced, suggesting that AhR activation resulted in either upregulation of posttranslation modification or downregulation of degradation of the receptor.

Uncleaved osteopontin (OPN) is associated with cell adhesion, facilitating osteoclast attachment to bone matrix (105). On the other hand, cleaved OPN is chemotactic for neutrophils and leukocytes. OPN knockout bone marrow cells showed reduced migratory capacity *in vivo* (80), and antibody blockade of the binding domain reduced cell migration as well as PTH-induced osteoclast bone resorption (106). TCDD exposure reproduced the effects of antibody blockade, with a similar inhibition of OPN-induced cell migration (80). Our group has shown that TCDD inhibits BMP-2-mediated bone regeneration and spine fusion in the rat, with only a partial recovery of fusion capacity after cessation of exposure for a period of four half-lives (*T*_1/2_ for TCDD is 19 days in the rat) (89).

Vogel et al. demonstrated upregulation of *CCL2* in the liver, thymus, kidney, adipose tissue, and heart of C57/BL6 mice injected with TCDD (107). Other *in vitro* studies in cell lines of various sources also suggest a direct correlation between CCL2 and TCDD exposure. Upregulation of CCL1, CXCL1, CXCL13, and CXCL8 (IL-8) has been observed as well (91, 108–110), while CCL5 was downregulated in mouse CD4+ T-cells *ex vivo* (111). The induction of IL-8 has been proposed to be as a result of NF-kB cross-talk through RelB activation (90, 91, 112). The upregulation of IL-8 and CCL2 by TCDD corresponds with findings of decreased expression in AhR-KO mice spleen (96). Notably, there was a 15-fold increase in *CCR2* (the receptor for CCL2) expression in AhR-KO mice. These mice also demonstrated a >2-fold increase in *CXCR5* and *CCL20* (96). This supports the notion that the hyperactivated AhR may act as a negative regulator of BMSC proliferation and trafficking.

### Dioxin-Like Compounds

Dioxin-like compounds include other polycyclic aromatic hydrocarbons, such as Benzo[a]pyrene (BaP), polychlorinated dibenzofurans, and non-ortho-substituted or mono-ortho-substituted pentachlorobiphenyls (PCBs). Scant evidence can be found on the effects of these dioxin-like compounds on bone healing or any associated chemokine alterations. PCB-118 (2,3′,4,4′,5-pentachlorobiphenyl) exposure correlates with lower BMD (84) and induction of osteoclastic activity *in vivo* (113). No studies have found any associations with chemokine expression changes and PCB-118. Three additional environmental dioxin-like toxicants, PCB-126 (3,3′,4,4′,5-pentachlorobiphenyl), PCB-77 (3,3′,4,4′-tetrachlorobiphenyl), and BaP, have been shown to upregulate expression of CCL2 and CXCL13 in both *in vitro* and *in vivo* models, the latter of which contributes to osteoblast development (114–119). BaP has also been shown to reduce *CCL5/RANTES* mRNA expression during osteoclast formation in human keratinocytes (118). Considering that CCL5 promotes osteoclast development and chemotaxis (44), further work is needed to identify any direct effects of BaP on CCL5 expression in developing osteoclasts. Table 3 reviews the changes in bone repair chemokines by TCDD and the other dioxin-like compounds found in current literature.

**Table 3:** **Dioxin and dioxin-like compounds: chemokine changes**.

Substance	Chemokine(s) involved	Effect(s)	Cell/tissue type	Reference
TCDD	CXCR4	↓ Migration		
		
	CXCL12	↑	Mouse HSC	Casado et al. (80)
		
	*CCL2*	↓		

		↑	Mouse thymus, liver, kidney, adipose, and cardiac tissue	Vogel et al. (107)

	CXCL1	↑	Mouse peritoneal B1 cells	Ishikawa (110)

	CXCL8		Human MCF-7	Monteiro et al. (108)

	CXCL12		Human synovial tissue	Kobayashi et al. (109)

	CXCL13		Mouse thymus, liver, kidney, adipose, and cardiac tissue	Vogel et al. (107)

	CCL1		Human MCF-7	

			Human synovial tissue	

	CCL5	↓	Mouse CD4+ T-cells	Marshall et al. (111)

PCB-126	CXCL8	↑	Porcine endothelial cells	Majkova et al. (115)
	CXCL13			
	CCL1		Human NHEK	Tsuji et al. (116)
	CCL2			
		
PCB-77			Primary human macrophages	N’Diaye et al. (117)
		
BaP			Human HaCaT and NHEK, mouse keratinocytes	Morino-Koga et al. (118)

BaP	*CCL5*	↓	Human HaCaT and NHEK, mouse keratinocytes	Morino-Koga et al. (118)

	*CXCL10* *CXCL9*	No change			

*Known effects of dioxin and dioxin-like compounds on chemokines (HSC, hematopoietic stem cells, TCDD, 2,3,7,8-tetrachlorodibenzo-*p*-dioxin; PCB-126, 3,3′,4,4′,5-pentachlorobiphenyl; PCB-77, 3,3′,4,4′-tetrachlorobiphenyl; BaP, benzo[a]pyrene)*.

**Figure 1:**
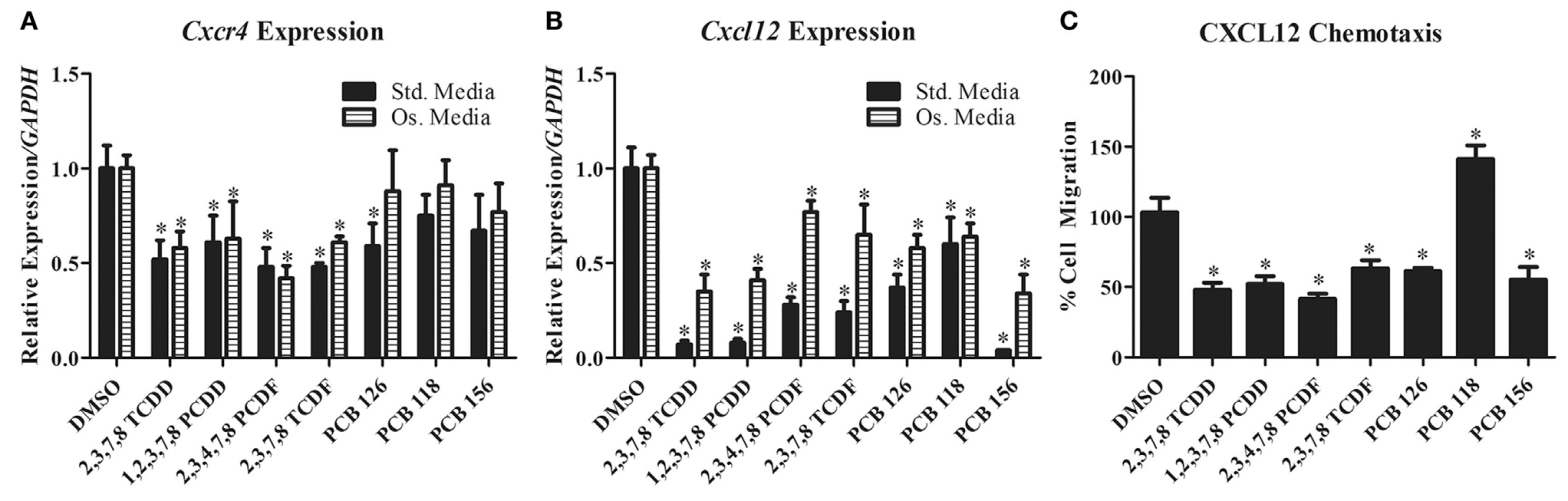
**(A,B)** *Cxcr4* and *Cxcl12* gene expression changes in primary rat BMSC grown in either standard or osteogenic media and treated with TCDD and dioxin-like compounds. **(C)** Chemotaxis rate of BMSC (toward 200 ng/mL of recombinant CXCL12) pretreated with TCDD and dioxin-like compounds. *p < 0.05 relative to DMSO vehicle control-treated cells. 2,3,7,8-TCDD, 2,3,7,8-tetrachlorodibenzo-*p*-dioxin; 1,2,3,7,8-PCDD, 1,2,3,7,8-pentachlorodibenzo-*p*-dioxin; 2,3,4,7,8-PCDD, 2,3,4,7,8-petachlorodibenzofuran; 2,3,7,8-TCDF, 2,3,7,8-tetrachlorodibenzofuran; PCB-126, 3,3′,4,4′,5-pentachlorobiphenyl; PCB-118, 2,3′,4,4′,5-pentachlorociphenyl; PCB-156, 2,3,3′,4,4′,5-hexachlorobiphenyl.

We previously found that TCDD exposure inhibits spine fusion in rats (89). Subsequent work has shown that similar to breast and ovarian cancer cells, TCDD downregulates CXCL12 and CXCR4 expression in differentiating primary rat BMSC (our own unpublished data and Figures 1A,B). Concordant with this, BMSC chemotaxis toward CXCL12 was also reduced after TCDD treatment (unpublished data and Figure 1C). Since no such studies have investigated the effects of dioxin-like compounds on the CXCR4/CXCL12 axis, we performed similar work to examine the effects of these compounds on CXCL12 and CXCR4 expression in primary rat BMSC grown in both standard and osteogenic conditions.

A select group of dioxins and dioxin-like compounds were chosen based on representative classification (dioxin, furan, or PCB) as well as the compound’s toxic equivalence within its class according to the World Health Organization (120). Northwestern University Institutional Animal Care and Use Committee (IACUC) approval was obtained prior to animal procedures and bone marrow stromal cell harvest. All experimental procedures were conducted in accordance with the recommendations of the IACUC. Rat BMSC were treated under standard or osteogenic conditions with various concentrations determined to activate the Ahr without inducing cell death (Table S1 in Supplementary Material). Total RNA was isolated using TRIzol (Invitrogen) and mRNA expression of *CXCL12* and *CXCR4* were analyzed using qPCR (Figures 1A,B). For chemotaxis assays, cells were counted by three blinded, independent reviewers (Figure 1C). Groups were compared using ANOVA and unpaired *t*-tests *post hoc*, with significance of *p* < 0.05.

*CXCR4* expression was significantly decreased in primary rat BMSC grown under both standard and osteogenic conditions for all but three of the treatment groups (PCB-118, -126, and -156). PCB-126 showed a significant decrease of *CXCR4* expression only under standard conditions. PCB-118 and -156 showed no changes in expression in either conditions. *CXCL12* was significantly reduced for all treatment groups in both conditions. Additionally, we evaluated the capacity of BMSC to migrate toward a CXCL12 gradient after treatment with these compounds. Chemotaxis was significantly reduced in cells pretreated with all but PCB-118, which was likely a result of a lack of *CXCR4* downregulation by PCB-118. Interestingly however, PCB-156 had no significant impact on *CXCR4* expression, yet CXCL12-induced chemotaxis was significantly decreased.

There is a growing appreciation for the deleterious effects of dioxin-like compounds on bone. As the physiologic role of the AhR pathway becomes better understood, so too will AhR-mediated changes in chemotactic signaling. Further research is prudent to better understand the mechanisms of dioxin-induced inhibition of bone healing.

## Metals

Another major source of chemokine-altering factors is circulating metal ions. Metal ions such as Mg, Fe, Cu, Zn, Mn, and Co are crucial for normal cellular functions but are toxic at elevated levels (121). These and other more exotic metals are being more keenly studied under the context of the progressive wear of metal prosthetic implants as well as other sources of environmental exposure.

### Environmental Metals

Environmental metal pollution has long been a topic of interest to toxicologists, given the growth of industry and technology, which has led to supraphysiologic metal exposure in humans. Of particular interest are metals that are commonly encountered in developed or developing nations through food, drinking water, air, and soil. Lead, cadmium, strontium, and lithium have all been shown to affect levels of chemokines related to bone healing. Tungsten, arsenic, iron, boron, and mercury have been shown to impact bone homeostasis and healing potential, albeit with lacking evidence of alterations in chemokine expression levels (Table 4).

**Table 4:** **Environmental metals: chemokine changes**.

Substance	Chemokine(s) involved	Effect(s)	Cell/tissue type	Reference
Lead	*CXCL12*	↓	*In vivo* mouse model	Beier et al. (122)

Cadmium	CXCL1	↑	Mouse RAW 264.7 Macrophages	Riemschneider et al. (123)

	CXCL8	↑	Human THP-1	Freitas and Fernandes (124)

Lithium	CXCL4	↑	Human mesenchymal stem cell (MSC)	Satija et al. (125)
		
	CXCR12		Human PBMC and PMN	Kim et al. (126)

	CXCL8	↓	Human MSC	Satija et al. (125)
		
	CCL20		Human PBMC and PMN	Kim et al. (126)

Strontium	CXCL8	↓	Human primary monocytes	Buache et al. (127)

*Known effects of environmental metals on chemokines*.

#### Lead

Despite the discovery of elevated lead levels in the drinking water in Flint, Michigan and other areas, the rate of lead toxicity as a result of environmental exposure has drastically improved since the 1970s. At that time, three-fourths of Americans had blood lead levels above 10 μg/dL, which had been the upper limit for categorization as lead poisoning (128, 129). Today, lead levels above 5 μg/dL warrant concern, according to the 2012 CDC update (130). Given the fact that bone acts as the main repository for lead (90–95%) and the body burden persists throughout life (lead *t*^1/2^ = 20 years), an extensive amount of research has evaluated its impact on bone health (131). Both basic and clinical studies suggest that lead has a significant impact on both bone growth/development and mature bone homeostasis. Similarly, lead exposure correlates with greater fracture risk and bone disease (25, 132, 133). Thus far, a dose-dependent response to lead accumulation in bone has been observed, causing osteopenia and poor BMD (133–136). However, it has been observed that low levels of lead cause increased BMD. The threshold that dictates the shift from increasing BMD to decreased BMD is still unknown (122, 137).

The only known study to have investigated chemokine fluctuations with exposure to lead is mouse tibia fracture study by Beier et al. This group found a ~50% reduction in *CXCL12* mRNA expression 10 days postoperative, which was after 52 total days of lead exposure (15–18 μg/dL). Treatment with the GSK-3β inhibitor, 6-bromoindirubin-3′-oxime (BIO), rescued the inhibitory effect on CXCL12. BIO treatment also increased β-catenin staining to control levels (138), suggesting Wnt involvement. Indeed, BIO has been shown to induce CXCL12 expression in mouse tibial fracture callus (139). The Beier study suggests that lead may in part impact fracture repair by reducing CXCL12/CXCR4-mediated progenitor cell recruitment to the site of injury. No other group has attempted to investigate the mechanisms by which lead inhibits bone healing or alters chemokine expression.

#### Cadmium

Cadmium has long been recognized as a health hazard. It was originally described in Japan as Itai-Itai “ouch-ouch” disease in 1955, so named for the pain from osteomalacia and frequent long bone fractures, which occur secondarily to the ingestion of cadmium-contaminated rice (140). Today, the greatest source of cadmium comes from food (cereals/vegetables/potatoes) and tobacco smoke (141, 142). The toxicity of cadmium is in part due to its resemblance to metals such as calcium, iron, and zinc. Early kidney damage and osteoporosis have been the most widely studied consequences of cadmium exposure (141, 143). Long-term environmental exposure has been shown to cause osteoporosis with subsequent increases in fragility fractures (144–148).

Cadmium-induced reduction in BMD has been linked to both renal proximal tubule damage, osteoblast toxicity, and stimulation of osteoclastic bone resorption. Very few studies have attempted to uncover the direct mechanisms of cadmium-induced bone loss. Thus far, cadmium exposure has been shown to increase RANKL production with no change in OPG, increase prostaglandin E2, and trigger apoptosis in osteoblasts (149, 150).

In terms of chemokines and chemotaxis, Riemschneider et al. reported a twofold increase in secretion of CXCL1 in macrophage RAW 264.7 cells with exposure to 10 µM Cadmium. Similarly, they found reduced levels of IL-6 and IL-10 (123). Cadmium-mediated activation of NF-kB has also been reported, with a subsequent increase in IL-8, IL-6, IL-1β, and TNF-α (124). Together, this pattern suggests increased recruitment of preosteoclastic cells, which may contribute to observed decreases in BMD. Similarly, bone regeneration may be negatively affected by a dysregulated inflammatory response, since inflammatory signaling has a crucial role in optimal bone healing (151). Furthermore, Papa et al. showed that cadmium induced the destruction of the osteoblast cytoskeleton actin network, which is critical for cell polarity, motility, and chemotaxis (152). The actin depolymerization that is seen with cadmium exposure may also affect osteoblastic chemotaxis *via* the CXCL12/CXCR4 axis, which again is crucial in early bone formation (153, 154). Further research should be directed toward elucidating the mechanisms by which cadmium alters the chemotactic responses of osteoblasts/osteoclasts.

#### Lithium

Lithium has long been used as a psychoactive drug in the treatment of various mood disorders. Recently, lithium has been recognized with growing concern as an environmental contaminant, due to greatly increased use of lithium ion batteries and alloys by industry and consumers. The most significant source of non-medical human exposure to lithium is thought to be through drinking water (155–157).

The effects of lithium on osteogenic differentiation vary according to cell system, state of differentiation, medium composition, passage number, and cell density (125). Lithium has been shown to inhibit Smad 1/5/8 phosphorylation in MC3T3-E1 preosteoblasts and murine myoblastic C2C12 cells, with a subsequent reduction in BMP-2-induced ALP activity independent of Wnt signaling (158). On the other hand, numerous studies have shown that lithium exposure induces a pro-bone formation phenotype, originating from osteoblastic activation and osteoclastic inhibition (159–163), with simultaneous antiadipogenic effects (125). Generally, Wnt/β-catenin pathway activation has been linked to most of the direct effects of lithium on bone homeostasis. Lithium inhibits GSK-3β, thereby preventing degradation of β-catenin (164). Enhanced nuclear β-catenin activity favors osteogenic differentiation of BMSC, reduced CXCL12 expression, and increased ALP activity in human MSC, although ALP activity was reduced relative to controls at higher doses (125). Expression levels of the pro-osteoclastic chemokines, *IL-7, IL-8*, and *CCL20* were also reduced after hMSC exposure to lithium (125).

In the context of bone healing under chronic environmental lithium exposure, dysregulation of Wnt signaling—tight temporal control of which is critical for osteogenesis to proceed normally—may disturb the natural exit from the proliferative phase and completion of the differentiation program (165). Still, it is possible that precisely timed administration of lithium may promote osteoblastogenesis and inhibit osteoclastogenesis to result in overall improved bone healing (166). Further research into dosing and timing of lithium administration is required to validate lithium as a novel therapy for bone regeneration.

#### Strontium

Strontium appears to prevent the age-related transition of BMSC lineage commitment from osteoblasts to adipocytes *via* NFATc/Maf and Wnt signaling (167). It is also being used as a therapeutic agent to prevent postmenapausal osteoporosis (168, 169). The only investigation of strontium-mediated chemokine changes was conducted by Bauche et al., in which LPS-stimulated monocytes exposed to strontium revealed diminished levels of IL-8 production. They also found IL-6 and TNF-α levels to be reduced, suggesting that strontium may have anti-inflammatory properties in addition to a potential osteoblastic lineage redirection (127).

#### Iron

Excess iron has been shown to generate reactive oxygen species (ROS) *via* the Fenton reaction (170). In addition to the potential ROS-induced cytotoxicity, ROS have been shown to antagonize Wnt signaling in osteoblast precursors by utilizing the limited pool of β-catenin for FoxO transcription, rather than of T-cell factor-mediated transcription (171). This may in turn lead to decreased bone formation, although this theory has yet to be examined in an *in vivo* model. Other evidence suggests that iron-ROS trigger osteoclastic bone resorption (172), which may increase the risk of osteoporosis (173). Despite these links to adverse effects on bone, modification of chemokine expression by excess iron has not been investigated.

#### Other Environmental Metals

Minimal research has been conducted on the effects of tungsten, arsenic, strontium, or mercury on bone; even less attention has been paid to chemokine expression changes. Tungsten has been shown to inhibit osteoblast differentiation of MSC *in vitro* (174). Arsenic was also shown to inhibit differentiation of osteoblasts, which occurred *via* ERK signaling. *In vivo*, arsenic exposure resulted in decreased BMD and altered bone microstructure in the rat (174, 175). Exposure to mercury occurs primarily through human consumption of fish (176), which has been shown to cause decreased activity of osteoclasts, with little, if any increased activity of osteoblasts (177). Some studies have suggested that high blood levels of mercury may in fact lower the risk of postmenopausal osteoporosis (178, 179). However, we found no studies investigating mercury’s effects on chemokine expression. Further research is needed to better understand the mechanisms of action of these toxicants.

### Prosthetic Wear Particles

Aseptic periprosthetic osteolysis is one of the most common causes of long-term prosthetic joint failure. Reports have cited failure rates as high as 56% at 6.5 years postoperative (180–182). Constant friction through the joint surface of metal-on-metal (MoM) and polyethylene-on-metal prostheses generates non-biodegradable particulate debris. In addition to polyethylene particles, metal ions such as titanium (Ti), titanium-6%/aluminum-4%/vanadium (Ti6Al4V) alloy, zirconium (Zr) oxide, Zr alloy, cobalt, cobalt chrome alloy, cobalt nickel chrome alloy, and cobalt chrome molybdenum (CoCrMo) alloy are produced from wear. Elevated concentrations of these metals have been measured in periprosthetic tissue, serum, urine, and in distant organs (liver, lymph nodes, spleen) (183–186).

Metal-on-metal implants became very popular in the late 1980s, with over one million implanted in the US to date (187). Because they are thought to deteriorate more slowly, MoM implants are used increasingly in young patients. However, elevated serum levels of metal ions, adverse reactions to metal debris, aseptic lymphocyte-dominated vasculitis, pseudotumors, and metal hypersensitivity are thought to lead to early loosening (188–199).

Metal-on-metal implants were initially thought to cause an immune reaction solely through the macrophage response; however, recent studies suggest that systemic metal ions, organometallic protein complexes, and particulate debris can also be immunoreactive (200). The mechanisms leading to pathologic bone resorption surrounding MoM implants are poorly understood. Numerous inflammatory cytokines and chemokines have been shown to be upregulated in peri-implant tissues of these patients, leading to a state of chronic inflammation and osteoclast activation/proliferation (201). Drynda et al. sought to clarify how circulating metal ions affect the CXCL12/CXCR4 axis and determine the impact this may have in BMSC homing/differentiation. Cultures of MG-63 (early-stage) and SaOs-2 (late-stage) osteoblast-like cells were exposed to Cobalt (Co), CoNiCrMo alloy (Nickel containing), or CoCrMo alloy (Nickel free). CXCL12/CXCR4 protein expression was dose-dependently activated in both cell lines with all metal exposures (202). Upregulation of CXCL12 in preosteoclasts has been associated with increased osteoclastic activity in several bone diseases, which may participate in eventual periprosthetic osteolysis (203–205). When AMD3100 was administered to block the interaction of CXCL12 with CXCR4, a partial reduction in TNF-α expression was observed. The same group also recovered periprosthetic tissue from patients undergoing revision for MoM aseptic loosening and found CXCR4 to be upregulated. Others have also reported similar increases in CXCR4 and TNF-α *in vivo* after exposure to Ti ions (206). Although no direct correlation can be drawn from either of these studies, it is possible that TNF-α may act as a trigger for CXCR4 upregulation.

Ti has been shown to increase CCL17 (TARC), CCL22 (MDC), and CCR4 expression (207). Cadosch et al. found that secretion of pro-inflammatory TNF-α, IL-6, and IL-1a/β was increased after Ti exposure, thereby leading to either direct osteoclast precursor activation or indirect activation through RANKL/M-CSF secretion by osteoblasts. RANKL activation also increased osteoclastic expression of CCL22 (208, 209), which lead to increased recruitment of CCR4^+^ osteoclast precursors. Correspondingly, there was an increase in CCL17 expression in hFOB 1.19 fetal osteoblastic cells and human osteoclasts. CCL17 functions in chemotactic recruitment of osteoclast precursors, likely through NF-kB activation (207).

Recent studies suggest that CCR4 is expressed in both Th2 and Th17 cells, and microscopic analysis of periprosthetic tissue reveals an observed increase in Th17 cell number (210). These periprosthetic Th17 cells may have been recruited and deposited through CCL22+ CCL17/CCR4-mediated chemotaxis and arrest (210). Furthermore, these deposited Th17 cells promote osteolysis through an IL-17-dependent increase in RANKL. Collectively, this is suggestive of a Ti-induced vicious cycle of osteoclastogenesis and inflammation (207).

Interestingly, the majority of Ti particle-induced osteolysis is associated with CXCR2, which is the receptor for IL-8 (211, 212). This receptor can be found on macrophages, osteoclasts, osteoblasts, and neutrophils (213), where ligand binding promotes neutrophil attraction, osteoclastic differentiation, and bone resorption (214). Silencing *CXCR2* mRNA reduced Ti-induced bone resorption rates in a mouse calvarial defect model by suppressing osteoclastogenesis indirectly through osteoblastic downregulation of RANKL (215). Several other groups have observed Ti-induced upregulation of CXCL8 (IL-8), CCL5 (RANTES), CCL3 (MIP-1α), and CCL2 (MCP-1) as a function of time-dependent NF-kB activation (216–220). Of these, IL-8 and CCL2 are strong chemoattractants for monocytes/macrophages/osteoclasts and neutrophils (221), which recruit bone-resorbing cells to induce periprosthetic osteolysis. Similar results were found with cobalt exposure in cultured human osteoblasts (222–224). Dalal et al. found that a CoCrMo alloy produced the greatest inflammatory response in comparison to Ti, Zr oxide, or Zr alloy, with a 100-fold increase (>2,000 pg/mL) of IL-8, a 30-fold increase of IL-6, and a 15-fold increase of TNF-α levels (224).

Metal particle-induced osteolysis is largely due to excessive osteoclast/inflammatory cell recruitment, osteoclastic activation, and inhibition of osteoblast activity. This occurs locally through phagocytosis and/or by the metal ions triggering toll-like receptors (214). These processes (is this true?) are mediated primarily by chemokines (225). Thus far, CXCL12/CXCR4, CCL17/CCL22/CCR4, IL-8/CXCR2, CCL5, CCL3, and CCL2 have all been implicated as mediators of prosthetic metal-induced bone destruction (Table 5). More research is needed to identify the chemokines responsible for recruitment of osteoclasts and Th17 cells, which in turn contribute to osteolysis and aseptic loosening. This understanding would provide for targeted approach to early diagnosis and treatment of prosthetic-induced loosening.

**Table 5:** **Prosthetic wear particles: chemokine changes**.

Substance	Chemokine(s) involved	Effect(s)	Cell/tissue type	Reference
Cobalt	CXCL4	↑	Human MG63 and SaOs-2	Drynda et al. (202)
	CXCR12			
CoNiCrMo alloy				
				
CoCrMo alloy				

Titanium	CXCR4		Rat tibia tissue	Omar et al. (206)
		
	CXCL8		Human periprosthetic granuloma tissue	Nakashima et al. (216)
	CCL3			
		
	CCR4	↑	Human fibroblasts	Trindade et al. (217)
		
	CCL5		Human MG63	Fritz et al. (219)
		
	CCL17 CCL22		Mouse GE-1 and MC3T3-E1	Wachi et al. (220)

*Known effects of prosthetic wear particles on chemokines (Co, Cobalt; Ni, Nickel; Cr, Chromium; Mo, Molybdenum)*.

## Other Environmental Factors of Interest

### Fluoride

Fluoride is found naturally and as an additive in tap water. It is well known for its ability to reduce the rate of dental caries. In low doses, fluoride increases osteoblast proliferation (226) and inhibits osteoclastic bone resorption (226, 227). Some clinical evidence suggests that fluoride may improve BMD (228), although other studies suggest that this more dense bone may in fact be more brittle and prone to fracture (226).

With high dose or prolonged exposure time, skeletal fluorosis and other toxic side effects may ensue. Skeletal fluorosis occurs secondarily to an increase in bone density, through a supraphysiologic increase of the growth of osteophytes. This results in symptoms and disability similar to what is observed in osteoarthritic patients. A safe level of fluoride in drinking water has proven difficult to establish. Many factors, including climate, air temperature, patient age, duration of exposure, and dietary calcium intake may play a role in the variability of doses reported in the literature, as each of these can alter fluoride bioavailability (229, 230). In an attempt to maintain safe doses of fluoride in drinking water, the EPA established and enforces a maximum allowable fluoride concentration of 4 mg/L; however, given recent experimental findings, the EPA now suggests maintaining fluoride levels at less than 2 mg/L to prevent possible deleterious effects.

Rat osteoblasts undergo apoptosis upon exposure to sodium fluoride (NaF) at doses of 500 µM and higher (231). At lower doses however, fluoride has positive effects on Wnt signaling, which leads to upregulation of osteoblastic differentiation makers, including ALP, COL1A1, osteonectin, and RunX2 in rat primary osteoblasts (232). Fluoride was also found to increase phosphorylation and inhibition of GSK-3β, resulting in prolonged activation of the Wnt pathway. Other groups have found *COL1A1* and *COL1A2* mRNA levels to be induced in rat osteoblasts after 24-h NaF exposure, although by 72 h, expression decreases in a dose-dependent fashion (231).

In addition to its direct effects on osteoblasts, fluoride has also been linked to anti-osteoclastic activity, with reduced cathepsin K, IL-1B, MMP-9, and TRAP activity upon RANKL- and M-CSF-induced osteoclastogenesis (233). In IL-1B-induced gingival inflammation, NaF was strongly anti-inflammatory, downregulating IL-1B, IL-8, and TNF-α expression at doses that did not induce apoptosis (234); however, the effects of fluoride on inflammation appear to depend on route of administration and are tissue specific (235–237). The inflammatory effects for fluoride in the context of bone have not been studied.

Fluoride may play a more direct role in calcium metabolism and bone turnover. In MC3T3E1 preosteoblasts, low-dose fluoride increased free calcium ion in the cell culture supernatant and inhibited PTH and PTHrP expression, with opposite effects on Ca++ and PTHrP at high NaF doses. *In vivo*, the same group found that NaF reduced serum calcium levels, and the effect of fluoride on PTHrP depended on whether the rats received standard or low calcium diet (238). These results suggest that high fluoride ingestion causes hypocalcemia by upregulating calcitonin through PTH and PTHrP secretion (238). Fluoride treatment also increased RANKL and OPG expression, hypothetically promoting osteoclast activation. Further research regarding chemokine signaling after fluoride exposure may explain the dose-dependent effects of fluoride on skeletal health.

### Molds and Fungal Toxins

Fungi and molds are common naturally occurring environmental contaminants. Toxic effects related to mold exposure are most commonly caused by mycotoxins and the various VOCs they produce. A study by Hokeness et al. evaluated the effects of two common VOCs (*(E)*-2-octenal and oct-1-en-3-ol) on BMSC viability (239). Both VOCs were cytotoxic, and the effect was determined to be secondary to alterations to membrane fluidity. The authors postulated that this could lead to breakdown of the immune system, but did not comment on the potential impact this could have on bone. However, membrane fluidity is known to affect osteoblastic differentiation of MSC (240).

The active compound from the mushroom *Cordyceps militaris*, cordycepin, is used medicinally for anti-inflammatory and chemotherapeutic purposes. Both *C. militaris* extract (CME) and isolated cordycepin have been shown to reduce RANKL-mediated osteoclastogenesis *in vitro*. In an *in vivo* murine model, cordycepin reduced LPS-induced inflammatory bone loss. Further studies will need to be conducted to determine whether cordycepin treatment is suitable in humans for the prevention of bone loss.

Peripheral blood mononuclear cells from mold-exposed workers expressed higher levels of eotaxin, INF-α, IL-1α, IL-12 p40, IL-12 p70, IP-10, PDGF-AA, TNF-β, and VEGF when exposed to a panel of common mold toxins *ex vivo* relative to cells from control patients (241). Asthmatic patients had a significant difference in chemokine expression, regardless of mold exposure. When exposed to aflatoxin B1 *in vitro*, polymorphonuclear leukocytes harvested from peripheral blood demonstrated decreased IL-8, CXCL1, and CXCL2 (242). While these studies demonstrate a link between exposure to fungal toxins and chemokines, it is unclear whether this has an impact on bone health. Subsequent research will be necessary to determine if fungal extracts and toxins are related to bone regeneration through chemokine mediation.

### Asbestos

Asbestos has a well-established role in inflammation and disease of respiratory tissues (243). Asbestos-derived ROS have been shown to activate TGF-β in lung epithelial tissue (244). CXCR3 levels have been reported to be decreased in CD4+ T-cells, inhibiting chemotaxis and potentially antitumor immunity in patients with asbestos-related lung disease (245). Other systemic markers of inflammation including IgE, IgA, IL-6, IL-8, and ICAM-1 have also been found to be elevated in asbestos-exposed persons (246). Upregulation of gremlin plays a role in asbestos-induced pulmonary fibrosis, which has been linked to decreased BMP and increased TGF-β signaling locally (247). No literature connecting asbestos to BMP, IL-8, or other chemokine levels in the context of bone were found. Further studies will be needed to determine if asbestos affects growth factor signaling in the bone, or has any impact on bone-related chemokines.

### Chlorine

While chlorinated compounds have been studied, no research examining the relationship between chlorine itself and bone-related chemokines has been conducted. Chlorinated and fluorinated hydroxyapatite-based biomaterials are currently under development. The addition of chlorine and fluorine to these scaffolds creates an acidic environment that simulates the osteoclastic conditions necessary for bone resorption and remodeling while simultaneously increasing apatite formation *in vitro* (248). Further studies will be necessary to determine if chlorine impacts bone healing through chemokine modulation, which may enhance or inhibit the osseointegration of chlorinated biomaterials.

## Conclusion

Environmental contaminants are ubiquitous in today’s world. Over the last several decades, public awareness that consumer goods are not necessarily safe has grown exponentially. Unfortunately, toxicological research cannot possibly keep pace with the rate at which new compounds are introduced into consumer goods, and a higher level of attention to the issue is prudent. Relatively little consideration has been given to the effects of environmental contaminants on bone and other musculoskeletal tissues, with even less focus on mechanisms of action. Considering the scale and impact of musculoskeletal disease and disorders on global health as well as the associated financial burden on the healthcare system, the MSK system should be a subject of major mechanistic toxicological research efforts.

There is a paucity of literature on the relationship between the majority of the environmental compounds reviewed here and chemokines relevant to bone. Many of the compounds discussed in this review warrant further research. Given their direct effects on PPARγ and adipogenic differentiation, organotins are of particular interest. Another subgroup of EDCs, the phthalate esters, have been shown to influence CXCL1 and CCL2 in other tissues. Given the known roles of CXCL1 and CCL2 in osteoclastogenesis, this could prove a high yield topic of study. The effect of prosthetic metals and periprosthetic wear particles may have on chemokines and bone regeneration is growing in clinical importance and represents another area of future focus. Finally, research on the effects of dioxins and dioxin-like compounds on bone-related chemokines will also continue to be a critical area of study, as their presence in cigarette smoke impacts such a large population.

The important roles that chemokines play in bone homeostasis and repair has become clear. Several therapeutics targeting chemokine receptors are now FDA-approved, albeit outside the context of MSK disease (1). A much more thorough understanding of the mechanisms by which environmental toxins adversely affect bone is critical to developing more effective, targeted approaches to mitigate these effects.

## Author Contributions

JS designed, drafted, edited manuscript, and interpreted basic science work. AS designed, drafted, edited manuscript, and interpreted basic science work. KK designed, drafted, edited manuscript, and interpreted basic science work. CY designed, performed original basic science work, and interpreted results. EH did experimental design and data interpretation and designed, directed, and edited manuscript.

## Conflict of Interest Statement

EH reports: consulting (immediate family member): Stryker, Bacterin, Graftys, Globus, AONA, Synthes, Spinesmith, SI Bone, Relievant, Ceramtec, Medtronic, Pioneer, Bioventus, and LifeNet. Speaking and/or teaching arrangements (immediate family member): AONA. Trips/trave (immediate family member): Stryker, Pioneer Surgical, Medtronic, Bioventus, and AONA. Board of Directors (immediate family member): Lumbar Spine Research Society and Cervical Spine Research Society. Scientific Advisory Board (immediate family member): Bioventus. JS, AS, KK, and CY report no relevant disclosures. None of the above disclosures are relevant to this paper. The authors declare that the research was conducted in the absence of any commercial or financial relationships that could be construed as a potential conflict of interest.

## Footnotes

^1^ Haas A. *Make No Bones About It: Environmental Contaminants Impact Bone Formation and the Immune System*. Available from: http://www.psr.org/chapters/boston/health-and-environment/make-no-bones-about-it.html.
